# Visual impairment and blindness caused by retinal diseases: A nationwide register-based study

**DOI:** 10.7189/jogh.13.04126

**Published:** 2023-11-03

**Authors:** Chuandi Zhou, Shu Li, Luyao Ye, Chong Chen, Shu Liu, Hongxia Yang, Peng Zhuang, Zengye Liu, Hongwen Jiang, Jing Han, Yiping Jiang, Liqin Zhou, Xiyuan Zhou, Jun Xiao, Cangxia Zhang, Lihui Wen, Changjun Lan, Yuqing Wang, Tao Sun, Li Jiang, Peipei Xie, Fan Chen, Ge Liang, Dongdong Fu, Tianzi Zhang, Xuetao Shi, Zhengyu Song, Xinghong Liu, Shanshan Li, Pengcheng Li, Xiangzhou Xu, Qinfeng Wei, Weibang Wang, Xionggao Huang, Zhen De, Aijun Deng, Lin Ding, Xiuhong Pan, Haiyan Wen, Zhongchen Zhang, Hongbin Lv, Jian Zhang, Xuemin Tian, Zhen Deng, Hua Wang, Fang Wang, Yu Wang, Hongchao Zhao, Yanhong Fang, Yuyu Wu, Yufeng Wu, Nian Shen, Bo Li, Xiaorong Li, Hong Dai, Naiqing Zhao, Xiaodong Sun, Zhi Zheng, Kun Liu, Xun Xu

**Affiliations:** 1 Department of Ophthalmology, Shanghai General Hospital, Shanghai Jiao Tong University School of Medicine, National Clinical Research Center for Eye Diseases, Shanghai Key Laboratory of Ocular Fundus Diseases, Shanghai Engineering Center for Visual Science and Photomedicine, Shanghai engineering center for precise diagnosis and treatment of eye diseases, Shanghai, China; 2 Department of Nursing, Shanghai General Hospital, Shanghai Jiao Tong University School of Medicine, Shanghai, China; 3 Department of Ophthalmology, Yantai Affiliated Hospital of Binzhou Medical University, Yantai, Shandong Province, China; 4 Department of Ophthalmology, The First People's Hospital of Jinzhong, Jinzhong, Liaoning Province, China; 5 Department of Ophthalmology, Zhangzhou Municipal Hospital of Fujian Province, Zhangzhou Affiliated Hospital of Fujian Medical University, Zhangzhou, Fujian Province, China; 6 Department of Ophthalmology, Tianjin First Central Hospital, Tianjin, China; 7 Department of Ophthalmology, The First People's Hospital of Kashgar, Kashgar, Xinjiang Uighur Autonomous Region, China; 8 Department of Ophthalmology, The Second Affiliated Hospital of Air Force Medical University, Xi'an, Shanxi Province, China; 9 Department of Ophthalmology, The First Affiliated Hospital of Gannan Medical University, Gannan, Jiangxi Province, China; 10 Department of Ophthalmology, The First Hospital of Jiaxing, Affiliatd Hospital of Jiaxing University, Jiaxing, Zhejiang Province, China; 11 Department of Ophthalmology, The Second Affiliated Hospital of Chongqing Medical University, Chongqing, China; 12 Department of Ophthalmology, The Second Hospital of Jilin University, Changchun, Jilin Province, China; 13 Department of Ophthalmology, Cangzhou Hospital of Integrated TCM-WM of Hebei, Cangzhou, Hebei Province, China; 14 Department of Ophthalmology, The Second People's Hospital of Guilin, Guilin, Guangxi Province, China; 15 Department of Ophthalmology, Affiliated Hospital of North Sichuan Medical College, Nanchong, Sichuan Province, China; 16 Department of Ophthalmology, The First Affiliated Hospital of Jiamusi University, Jiamusi, Heilongjiang Province, China; 17 Department of Ophthalmology, Yancheng City No. 1 People's Hospital, Yancheng, Jiangsu Province, China; 18 Department of Ophthalmology, Benxi Central Hospital, Benxi, Liaoning Province, China; 19 Department of Ophthalmology, The 152th Central Hospital of the People's Liberation Army, Pingdingshan, Henan Province, China; 20 Department of Ophthalmology, Anqing Municipal Hospital, Anqing, Anhui Province, China; 21 Department of Ophthalmology, PLA Rocket Force Characteristic Medical Center, Beijing, China; 22 Department of Ophthalmology, People's Hospital of Zunyi City Bo Zhou District, Zunyi, Guzhou Province, China; 23 Department of Ophthalmology, Affiliated Hospital of Inner Mongolia Minzu University, Tongliao, Inner Mongolia Autonomous Region, China; 24 Department of Ophthalmology, Pu’er City People's Hospital, Pu’er, Yunnan Province, China; 25 Department of Ophthalmology, Shuguang Hospital, Shanghai University of Traditional Chinese Medicine, Shanghai, China; 26 Department of Ophthalmology, The Central Hospital of Shaoyang, Shaoyang, Hunan Province, China; 27 Department of Ophthalmology, Workers' Hospital of Jinchuan Group Co., Ltd, Jinchang, Gansu Province, China; 28 Department of Ophthalmology, Union Hospital Tongji Medical College Huazhong University of Science and Technology, Wuhan, Hubei Province, China; 29 Department of Ophthalmology, Huizhou First Hospital, Huizhou, Guangdong Province, China; 30 Department of Ophthalmology, The Second People's Hospital of Xining, Xining, Qinghai Province, China; 31 Department of Ophthalmology, The First People's Hospital of Yinchuan, The Second Affiliated Hospital of Ningxia Medical School, Yinchuan, Ningxia Hui autonomous region, China; 32 Department of Ophthalmology, Hainan General Hospital, Hainan Affiliated Hospital of Hainan Medical University, Haikou, Hainan Province, China; 33 Department of Ophthalmology, Shigatse People's Hospital, Shigatse, Tibet Autonomous Region, China; 34 Department of Ophthalmology, Affiliated Hospital of Weifang Medical School, Weifang, Shandong Province, China; 35 Department of Ophthalmology, People's Hospital of Xinjiang Uygur Autonomous Region, Urumqi, Xinjiang Uygur Autonomous Region, China; 36 Yuncheng Eye Hospital, Yuncheng, Shanxi Province, China; 37 Department of Ophthalmology, People's Hospital of Jilin City, Jilin, Jilin Province, China; 38 Department of Ophthalmology, Hebei Yanda Hospital, Langfang, Hebei Province, China; 39 Department of Ophthalmology, The Affiliated Hospital of Southwest Medical University, Luzhou, Sichuan Province, China; 40 Department of Ophthalmology, Shaanxi Provincial People's Hospital, Xi’an, Shanxi Province, China; 41 Department of Ophthalmology, The 988th Hospital of Joint Logistics Support Force of the Chinese People's Liberation Army, Kaifeng, Henan Province, China; 42 Department of Ophthalmology, The First People's Hospital of Linping District, Hangzhou, Zhejiang Province, China; 43 Department of Ophthalmology, The First People's Hospital of Lianyungang, Lianyungang, Jiangsu Province, China; 44 Department of Ophthalmology, The Second Affiliated Hospital of Guizhou University of Traditional Chinese Medicine, Guiyang, Guizhou Province, China; 45 Fushun Ophthalmopathy Hospital, Fushun, Liaoning Province, China; 46 Department of Ophthalmology, People's Hospital of Yuxi City, Yuxi, Yunnan Province, China; 47 Department of Ophthalmology, Jiangjin Central Hospital, Chongqing, China; 48 Department of Ophthalmology, The Second Affiliated Hospital of Fujian Medical University, Quanzhou, Fujian Province, China; 49 Department of Ophthalmology, Qingyang People's Hospital, Qingyang, Gansu Province, China; 50 Department of Ophthalmology, Liuzhou People's Hospital, Liuzhou, Guangxi Zhuang Autonomous Region, China; 51 Department of Ophthalmology, Tianjin Medical University Eye Hospital, School of Optometry & Eye Institute, Tianjin, China; 52 Department of Ophthalmology, Beijing Hospital, Beijing, China; 53 Department of Biostatistics, Fudan University School of Public Health, Shanghai, China

## Abstract

**Background:**

Retinal disorders cause substantial visual burden globally. Accurate estimates of the vision loss due to retinal diseases are pivotal to inform optimal eye health care planning and allocation of medical resources. The purpose of this study is to describe the proportion of visual impairment and blindness caused by major retinal diseases in China.

**Methods:**

A nationwide register-based study of vitreoretinal disease covering all 31 provinces (51 treating centres) of mainland China. A total of 28 320 adults diagnosed with retinal diseases were included. Participants underwent standardised ocular examinations, which included best-corrected visual acuity (BCVA), dilated-fundus assessments, and optical coherence tomography. Visual impairment and blindness are defined using BCVA according to the World Health Organization (WHO) (visual impairment: <20/63-≥20/400; blindness: <20/400) and the United States (visual impairment: <20/40-≥20/200; blindness: <20/200) definitions. The risk factors of vision loss were explored by logistic regression analyses.

**Results:**

Based on the WHO definitions, the proportions for unilateral visual impairment and blindness were 46% and 18%, respectively, whereas those for bilateral visual impairment and blindness were 31% and 3.3%, respectively. Diabetic retinopathy (DR) accounts for the largest proportion of patients with visual impairment (unilateral visual impairment: 32%, bilateral visual impairment: 60%) and blindness (unilateral blindness: 35%; bilateral blindness: 64%). Other retinal diseases that contributed significantly to vision loss included age-related macular degeneration, myopic maculopathy, retinal vein occlusion, and rhegmatogenous retinal detachment and other macular diseases. Women (bilateral vision loss: *P* = 0.011), aged patients (unilateral vision loss: 45-64 years: *P* < 0.001, ≥65 years: *P* < 0.001; bilateral vision loss: 45-64 years: *P* = 0.003, ≥65 years: *P* < 0.001 (reference: 18-44 years)) and those from Midwest China (unilateral and bilateral vision loss: both *P* < 0.001) were more likely to suffer from vision loss.

**Conclusions:**

Retinal disorders cause substantial visual burden among patients with retinal diseases in China. DR, the predominant retinal disease, is accountable for the most prevalent visual disabilities. Better control of diabetes and scaled-up screenings are warranted to prevent DR. Specific attention should be paid to women, aged patients, and less developed regions.

Visual impairment and blindness cause substantial functional and social limitations [1-3], and preventing visual disabilities is a major goal of eye care services. In 2020, approximately 43.3 million people are blind globally, and 553 million have varying degrees of visual impairment [4], and as much as 64% of them are in Asia [5].

The causes of vision loss vary greatly among different regions [1,5]. Apart from cataract and refractive error, retinal diseases remain significant contributors [1,5-7]. The most frequent retinal disorders attributed to vision loss included diabetic retinopathy (DR), age-related macular degeneration (AMD), myopic maculopathy, retinal vein occlusion (RVO), retinal detachment, and et al. Unlike completely curable causes, the vision loss caused by retinal diseases is hard to reverse. Therefore, prevention, early detection and timely treatment of retinal abnormalities may be more beneficial. On the other hand, in the past few decades, significant advances in diagnosis, new drugs and surgical techniques have revolutionised the prognosis of retinal diseases. Therefore, a contemporary update on the visual burden caused by retinal diseases is warranted for optimal policy planning and allocation of medical resources.

DR and AMD have been among the top five causes of vision loss globally [1,5]. In addition, myopic maculopathy, with a rising prevalence worldwide, has caused a severe visual burden particularly in East Asia and Europe [8-10]. Moreover, RVO, which exhibits the highest prevalence in Asian and Hispanics, is also a frequent cause of vision loss [11,12]. And other common retinal diseases, such as rhegmatogenous retinal detachment (RRD), macular hole, retinal pigmentosa and et al., also result in varying degrees of visual impairment. China, as the most populous country in the world, has a fifth of the global population. With the rapidly growing numbers of ageing population and diabetes, visual impairment due to AMD, DR and other chronic retinal diseases may also have elevated. Nevertheless, the contribution to visual disabilities by major types of retinal diseases has not been assessed on a national scale.

To our knowledge, most of the previous studies explored vision loss through population-based studies. However, in these studies, cataract accounts for a substantial proportion of visual impairment, in which, lens opacity precludes identifying retinal diseases. And most of studies did not include detailed dilated-fundus assessments. All these factors might have underestimated retinal diseases. Moreover, prior population-based studies only had a small number of patients in the subset with retinal disorders [13-15], which may induce a bias in assessing its impact on vision. However, in most large studies, the retinal disease type was not specified or only included very limited number of retinal abnormalities [7,16,17]. Therefore, a comprehensive evaluation of visual disabilities caused by retinal diseases needs to be assessed in a large sample including a wide spectrum of retinal disorders on the basis of detailed fundus examinations.

In this multicenter study covering all 31 provinces of mainland China, we analysed best-corrected visual acuity (BCVA) of 28 320 adults with various retinal disorders from 51 medical centres, to estimate the proportion of visual impairment and blindness among patients with retinal disorders.

## METHODS

### Study design and procedures

A nationwide registration of vitreoretinal disease (Official website: http://www.brightnesscenter.com/) was constructed by National Clinical Research Center for Eye Diseases from April 2020 based on personal interactions, linking to professional societies and communications at scientific conferences. Our system included an expert panel, an academic committee and an independent panel for quality control. This consortium has expanded to 51 treating centres covering all 31 provinces of mainland China till now. Treating centres, at least could perform fundus OCT and intravitreal injection of anti-VEGF, were eligible to participate.

### Ocular examinations and data collection

This study adhered to the tenets of the Declaration of Helsinki. A centralised Institutional Review Boards (IRB) Review Process was applied in this multicentre study. This study was approved by the IRB of the lead unit, Shanghai General Hospital (No. 2022-KY-021). Informed consent was obtained from all patients.

Consecutive patients who were diagnosed with retinal diseases from April 2020 to November 2021 were included. The exclusion criteria were as follows: (1) incomplete data collection for major parameters; (2) younger than 18 years.

All practitioners were trained before joining this programme using unified protocols. They collected and input the data to the same system. At each study site, all patients underwent standardised ocular examinations, which included visual acuity (VA) testing, a slit-lamp and fundus examination before and after pupil dilation and fundus OCT (Heidelberg Engineering, Heidelberg, Germany). Other tests consisting of intraocular pressure (Canon Medical System, US), ocular B-ultrasonography (Aviso, Quantel Medical, France), fundus photographs (Neitz, Japan), fundus fluorescein angiography (FFA) (Heidelberg Engineering, Heidelberg, Germany), and indocyanine green angiography were performed if necessary. For each eye, the VA was measured with a logarithm E chart at a distance of five meters [18]. If no letters were recognised at five meters, the patient moved to four, three, two or one meter, consecutively. If no letters could be read on the chart, VA was assessed as counting fingers, hand movements, light perception, and no light perception. When the presenting VA was 20/30 or worse, BCVA, in which refraction was corrected by certified optometrists without cycloplegia, was documented. And all BCVA test was rechecked by a senior optometrist.

Two definitions of visual impairment and blindness, the United States (US) and the World Health Organization (WHO) criteria, were applied in this study. In the WHO definition, visual impairment and blindness are defined as BCVA<20/63-≥20/400 and BCVA<20/400, respectively. In the US definition, visual impairment and blindness mean BCVA<20/40-≥20/200 and BCVA<20/200, respectively. In addition to reporting the bilateral vision loss on the basis of the better-seeing eye among bilateral patients, we also documented the data on unilateral visual impairment, which was assessed in terms of the worse-seeing eye for the overall sample.

Data retrieved from medical charts included place of residence, contact information, laterality (unilateral/bilateral), gender, age, baseline BCVA, primary diagnosis of vision loss. Patients were subdivided using the age groups of 18 to 44 years, 45 to 64 years, and 65 years or older.

### Attribution of retinal causes of visual impairment and blindness

DR was graded according to the Arlie House classification system for the Early Treatment Diabetic Retinopathy Study [19] based on retinal photographs. AMD was assessed using the Wisconsin Age-related Maculopathy grading system [20]. Myopic maculopathy was defined as spherical equivalent of at least -6.0 dioptres with axial elongation and additional atrophic, tractional or neovascular changes [21]. The diagnosis of RVO is based on the fundus examination, aided by colour fundus photographs and/or FFA. Moreover, the diagnoses of other retinal diseases followed the clinical standard, such as RRD, epiretinal membrane, macular hole, central serous chorioretinopathy (CSC), hypertensive retinopathy, retinal pigmentosa, retinal artery occlusion (RAO), idiopathic choroidal neovascularisation (CNV), and vitreous macular traction. Of note, the relative rare retinal disorders, such as Coats disease, traumatic retinal diseases, and et al, which accounted for <0.1% of total sample, were grouped as other retinal disorders.

After data collection, a panel of senior investigators (KL, XX, ZZ, XL, HD) checked all retinal causes of visual impairment and blindness. After reviewing medical charts and imaging data, only one diagnosis was chosen as the primary cause. Nevertheless, when multiple disorders were present in one patient, the disorder causing the greatest visual limitation was identified as the primary cause. The clinical data of each patient was reviewed by at least two graders. The consistency between the results from the two graders was 98.9%. A third grader, who was not involved in the above assessment, was invited to deliberate discrepancies. However, the exact causes for 546 (1.9%) patients with vitreous haemorrhage and 675 (2.4%) with macular oedema could not be allocated.

### Statistical analysis

Statistical analysis was performed using IBM SPSS Statistics (version 24.0; Chicago, USA). Proportion estimates and 95% confidence intervals (CIs) of visual impairment and blindness were calculated by age, gender and geographic region. Univariate and multivariate logistic regressions were used to explore the risk factors of vision loss, including visual impairment and blindness. Odds ratios (ORs) and 95% CIs were documented. A *P* < 0.05 was considered statistically significant.

## RESULTS

### BCVA and visual impairment

A total of 28 320 patients aged 18 to 99 years were diagnosed with retinal disorders. The median age was 62 years (interquartile range: 53-70 years). The number of patients allocated into the age groups of 18 to 44 years, 45 to 64 years, and 65 years or older were 3177 (11%), 13 189 (47%) and 11 954 (42%), respectively. In this sample, 14 293 (50%) were men and 14 027 (50%) were women. The participants were almost evenly distributed in East (14 011, 49%) and Midwest (14 309, 51%) China.

The proportion of unilateral and bilateral visual impairment and blindness caused by major types of retinal diseases are summarised in **Table 1** and **Table 2**, respectively. By the WHO criteria, the percentage of unilateral visual impairment and blindness were 46% and 18%, respectively. On the basis of the US criteria, the proportion of unilateral visual impairment remained similar (45%), while that of blindness elevated (27%). A slightly higher frequency of unilateral visual impairment was observed in women (WHO criteria: 47 vs. 45%; US criteria: 47 vs. 44%) and those from East China (WHO criteria: 47 vs. 45%; US criteria: 46 vs. 44%) according to both criteria. In addition, the proportion of unilateral visual impairment and blindness elevated with age for both definitions.

**Table 1:** The proportion of unilateral visual impairment and blindness caused by retinal diseases

	Total	Visual impairment				Blindness			
		World Health Organization (BCVA, <20/63-≥20/400)		United States (BCVA, <20/40-≥20/200)		World Health Organization (BCVA, <20/400)		United States (BCVA, <20/200)	
	No.	No.	% (95% CI)	No.	% (95% CI)	No.	% (95% CI)	No.	% (95% CI)
Total	28 320	12 992	46 (45-46)	12 803	45 (45-46)	5046	18 (17-18)	7630	27 (26-27)
Gender									
*Male*	14 293	6421	45 (44-46)	6263	44 (43-45)	2601	18 (18-19)	3917	27 (27-28)
*Female*	14 027	6571	47 (46-48)	6540	47 (46-47)	2445	17 (17-18)	3713	26 (26-27)
Age									
*18-44 years*	3177	1152	36 (35-38)	1253	39 (38-41)	435	14 (12-15)	616	19 (18-21)
*45-64 years*	13 189	5826	44 (43-45)	5866	44 (44-45)	2258	17 (16-18)	3334	25 (25-26)
*≥65 years*	11 954	6014	50 (49-51)	5684	48 (47-48)	2353	20 (19-20)	3680	31 (30-32)
Region									
*East China*	14 011	6544	47 (46-48)	6506	46 (46-47)	2135	15 (15-16)	3409	24 (24-25)
*Midwest China*	14 309	6448	45 (44-46)	6297	44 (43-45)	2911	20 (20-21)	4221	29 (29-30)

BCVA – best-corrected visual acuity, CI – confidence interval

**Table 2:** The proportion of bilateral visual impairment and blindness caused by retinal diseases

	Total	Visual impairment				Blindness			
		World Health Organization (BCVA, <20/63-≥20/400)		United States (BCVA, <20/40-≥20/200)		World Health Organization (BCVA, <20/400)		United States (BCVA, <20/200)	
	No.	No.	% (95% CI)	No.	% (95% CI)	No.	% (95% CI)	No.	% (95% CI)
Total	9414	2872	31 (30-31)	3570	38 (37-39)	313	3.3 (3.0-3.7)	610	6.5 (6.0-7.0)
Gender									
*Male*	4749	1378	29 (28-30)	1707	36 (35-37)	150	3.2 (2.7-3.7)	289	6.1 (5.4-6.8)
*Female*	4665	1494	32 (31-33)	1863	40 (39-41)	163	3.5 (3.0-4.0)	321	6.9 (6.2-7.6)
Age									
18-44 *years*	1083	250	23 (21-26)	317	29 (27-32)	33	3.0 (2.0-4.1)	57	5.3 (3.9-6.6)
45-64 *years*	4477	1261	28 (27-29)	1590	36 (34-37)	140	3.1 (2.6-3.6)	262	5.9 (5.2-6.5)
≥65 *years*	3854	1361	35 (34-37)	1663	43 (42-45)	140	3.6 (3.0-4.2)	291	7.6 (6.7-8.4)
Region									
*East China*	4547	1304	29 (27-30)	1666	37 (35-38)	105	2.3 (1.9-2.7)	227	5.0 (4.4-5.6)
*Midwest China*	4867	1568	32 (31-34)	1904	39 (38-40)	208	4.3 (3.7-4.8)	383	7.9 (7.1-8.6)

BCVA – best-corrected visual acuity, CI – confidence interval

According to the WHO criteria, the percentage of bilateral visual impairment and blindness were 31% and 3.3%, respectively. The proportion of bilateral visual impairment (38%) and blindness (6.5%) were both elevated when using the US criteria. Women had a higher level of bilateral visual impairment (WHO criteria: 32 vs. 29%; US criteria: 40 vs. 36%) and blindness (WHO criteria: 3.5 vs. 3.2%; US criteria: 6.9 vs. 6.1%) than men. In addition, both bilateral visual impairment and blindness were more prevalent in Midwest China than in East China (WHO criteria: visual impairment: 32 vs. 29%; blindness: 4.3 vs. 2.3%; US criteria: visual impairment: 39 vs. 37%; blindness: 7.9 vs. 5.0%). Also, the proportion of both visual impairment and blindness increased steadily with age.

### Retinal causes of visual impairment and blindness

**Figure 1:**
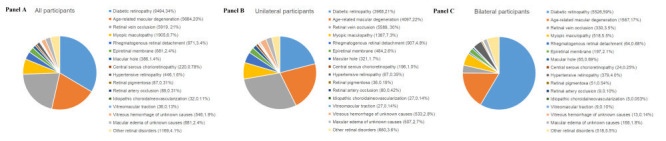
Pie charts demonstrating the distribution of retinal diseases in Chinese adults aged 18 to 99 years. **Panel A.** All participants (n = 28 320). **Panel B.** Participants with unilateral retinal diseases (n = 18 906). **Panel C**. Participants with bilateral retinal diseases (n = 9414).

Around two thirds of patients (18 906, 67%) had unilateral retinal diseases, and the remaining one-third (9414, 33%) were bilaterally involved. The top three retinal disorders were DR (9494, 34%), RVO (5919, 21%) and AMD (5664, 20%). Others included myopic maculopathy (1905, 6.7%), RRD (971, 3.4%), epiretinal membrane (681, 2.4%), macular hole (386, 1.4%), CSC (220, 0.78%), hypertensive retinopathy (446, 1.6%), retinal pigmentosa (87, 0.31%), RAO (89, 0.31%), idiopathic CNV (32, 0.11%), vitreous macular traction (36, 0.13%), vitreous hemorrhage of unknown causes (546, 1.9%), macular oedema of unknown causes (675, 2.4%), and other relatively rare retinal disorders (1169, 4.1%). Of note, the most prevalent unilateral retinal disease is RVO (5589, 30%), followed by AMD (4097, 22%), DR (3968, 21%), myopic maculopathy (1387, 7.3%) and RRD (907, 4.8%). For bilaterally involved retinal disturbances, DR (5526, 59%), AMD (1567, 17%), myopic maculopathy (518, 5.5%) and RVO (330, 3.5%) were the top four leading diseases. The detailed distribution of retinal diseases for the total sample, unilateral and bilateral patients is displayed in **Figure 1**, panels A, B and C.

The proportional retinal causes of unilateraland bilateral visual impairment and blindness are summarised in **Table 3** and **Table 4**, respectively. According to WHO criteria, DR causes the largest proportion of unilateral visual impairment, accounting for 32% and 35% of unilateral visual impairment and blindness, respectively. AMD (visual impairment: 23%, blindness: 20%) and RVO (visual impairment: 24%, blindness: 15%) were the second and third frequent causes of unilateral vision loss. Other contributing causes included myopic maculopathy (visual impairment: 7.1%, blindness: 5.6%), RRD (visual impairment: 2.7%, blindness: 7.2%), epiretinal membrane (visual impairment: 2.3%, blindness: 1.0%) and macular hole (visual impairment: 1.7%, blindness: 1.6%). Notably, the distributions of the causes of vision loss varied by gender and age. AMD, RRD and CSC were the more common causes of visual impairment and blindness in men than in women, with relative age-adjusted ORs of women vs. men of 0.70 (95% CI = 0.65-0.76) for AMD, 0.83 (95% CI = 0.71-0.96) for RRD, and 0.31 (95% CI = 0.16-0.61) for CSC. Whereas women were more likely to be blind or have visual impairment due to myopic maculopathy (OR = 1.73, 95% CI = 1.53-1.95) or epiretinal membrane (OR = 1.75, 95% CI = 1.40-2.18) or macular hole (OR = 2.18, 95% CI = 1.71-2.79) than men. Overall, the proportion of visual impairment and blindness increased with age, although the pattern of age-specific proportion varied by cause. Vision loss due to AMD, epiretinal membrane and macular hole increased with age, especially from age 45 years onward, although vision loss due to DR increased up to the age of around 64 years and decreased thereafter. Of note, myopic maculopathy, RRD and CSC affected adults with younger age. Similar trend was observed on the basis of US criteria (Table S1 and S2 in the **Online Supplementary Document**).

**Table 3:** Proportional retinal causes of unilateral visual impairment and blindness by age, gender and regions based on the World Health Organization criteria*

	Visual impairment (BCVA, <20/63-≥20/400)								Blindness (BCVA, <20/400)								
	**Total**	**Gender**		**Age (years)**			**Regions**		**Total**	**Gender**		**Age (years)**			**Regions**		
**Retinal diseases**		**Male**	**Female**	**18-44**	**45-64**	**≥65**	**Eastern**	**Midwest**		**Male**	**Female**	**18-44**	**45-64**	**≥65**	**Eastern**	**Midwest**
	n = 12 992	n = 6421	n = 6571	n = 1152	n = 5826	n = 6014	n = 6544	n = 6448	n = 5046	n = 2601	n = 2445	n = 435	n = 2258	n = 2353	n = 2135	n = 2911
DR	4222 (32)	2115 (33)	2107 (32)	402 (35)	2347 (40)	1473 (24)	2264 (35)	1958 (30)	1748 (35)	885 (34)	863 (35)	176 (40)	1043 (46)	529 (22)	790 (37)	958 (33)
AMD	3001 (23)	1621 (25)	1380 (21)	0 (0)	750 (13)	2251 (37)	1558 (24)	1443 (22)	1032 (20)	574 (22)	458 (19)	0 (0)	173 (7.7)	859 (37)	450 (21)	582 (20)
RVO	3104 (24)	1514 (24)	1590 (24)	250 (22)	1576 (27)	1278 (21)	1560 (24)	1544 (24)	737 (15)	388 (15)	349 (14)	48 (11)	284 (13)	405 (17)	305 (14)	432 (15)
Myopic maculopathy	920 (7.1)	356 (5.5)	564 (8.6)	260 (23)	411 (7.1)	249 (4.1)	375 (5.7)	545 (8.5)	283 (5.6)	125 (4.8)	158 (6.5)	43 (9.9)	133 (5.9)	107 (4.5)	115 (5.4)	168 (5.8)
RRD	348 (2.7)	193 (3.0)	155 (2.4)	79 (6.9)	189 (3.2)	80 (1.3)	150 (2.3)	198 (3.1)	365 (7.2)	211 (8.1)	154 (6.3)	66 (15)	214 (9.5)	85 (3.6)	130 (6.1)	235 (8.1)
ERM	294 (2.3)	104 (1.6)	190 (2.9)	5 (0.43)	76 (1.3)	213 (3.5)	133 (2.0)	161 (2.5)	51 (1.0)	20 (0.77)	31 (1.3)	1 (0.23)	14 (0.62)	36 (1.5)	17 (0.80)	34 (1.2)
Macular hole	224 (1.7)	73 (1.1)	151 (2.3)	11 (0.95)	98 (1.7)	115 (1.9)	122 (1.9)	102 (1.6)	81 (1.6)	23 (0.88)	58 (2.4)	4 (0.92)	39 (1.7)	38 (1.6)	38 (1.8)	43 (1.5)
CSC	48 (0.37)	38 (0.59)	10 (0.15)	19 (1.6)	24 (0.41)	5 (0.083)	22 (0.34)	24 (0.40)	4 (0.079)	3 (0.12)	1 (0.041)	1 (0.23)	3 (0.13)	0 (0)	1 (0.047)	3 (0.10)
Hypertensive retinopathy	57 (0.44)	33 (0.51)	24 (0.37)	9 (0.78)	16 (0.27)	32 (0.53)	30 (0.46)	27 (0.42)	20 (0.40)	12 (0.46)	8 (0.33)	3 (0.69)	7 (0.31)	10 (0.42)	13 (0.61)	7 (0.24)
RP	20 (0.15)	15 (0.23)	5 (0.076)	9 (0.78)	6 (0.10)	5 (0.083)	8 (0.12)	12 (0.19)	20 (0.40)	9 (0.35)	11 (0.45)	4 (0.92)	13 (0.58)	3 (0.13)	7 (0.33)	13 (0.45)
RAO	13 (0.10)	9 (0.14)	4 (0.061)	1 (0.087)	6 (0.10)	6 (0.10)	3 (0.046)	10 (0.16)	56 (1.1)	36 (1.4)	20 (0.82)	2 (0.46)	29 (1.3)	25 (1.1)	29 (1.4)	27 (0.93)
Idiopathic CNV	12 (0.092)	3 (0.047)	9 (0.14)	7 (0.61)	4 (0.069)	1 (0.017)	1 (0.015)	11 (0.17)	4 (0.079)	2 (0.077)	2 (0.082)	2 (0.46)	1 (0.044)	1 (0.042)	1 (0.047)	3 (0.10)
VMT	18 (0.14)	8 (0.12)	10 (0.15)	1 (0.087)	3 (0.051)	14 (0.23)	9 (0.14)	9 (0.14)	3 (0.059)	1 (0.038)	2 (0.082)	0 (0)	0 (0)	3 (0.13)	1 (0.047)	2 (0.069)
VH of unknown cause	134 (1.0)	67 (1.0)	67 (1.0)	14 (1.2)	75 (1.3)	45 (0.75)	57 (0.87)	77 (1.2)	311 (6.2)	163 (6.3)	148 (6.1)	24 (5.5)	165 (7.3)	122 (5.2)	135 (6.3)	176 (6.0)
ME of unknown cause	226 (1.7)	114 (1.8)	112 (1.7)	43 (3.7)	88 (1.5)	95 (1.6)	90 (1.4)	136 (2.1)	120 (2.4)	57 (2.2)	63 (2.6)	28 (6.4)	46 (2.0)	46 (2.0)	23 (1.1)	97 (3.3)
Others	351 (2.7)	158 (2.5)	193 (2.9)	42 (3.6)	157 (2.7)	152 (2.5)	162 (2.5)	189 (2.9)	211 (4.2)	92 (3.5)	119 (4.9)	33 (7.6)	94 (4.2)	84 (3.6)	80 (3.7)	13 1(4.5)

BCVA – best-corrected visual acuity, DR – diabetic retinopathy, AMD – age-related macular degeneration, RVO – retinal vein occlusion, RRD – rhegmatogenous retinal detachment, ERM – epiretinal membrane, CSC – central serous chorioretinopathy, RP – retinal pigmentosa, RAO – retinal artery occlusion, CNV – choroidal neovascularisation, VMT – vitreous macular traction, VH – vitreous hemorrhage, ME – macular edema

*The data was presented as number (proportion).

**Table 4:** Proportional retinal causes of bilateral visual impairment and blindness by age, gender and regions based on the World Health Organization criteria*

	Visual impairment (BCVA, <20/63-≥20/400)								Blindness (BCVA, <20/400)							
	**Total**	**Gender**		**Age (years)**			**Region**		**Total**	**Gender**		**Age (years)**			**Region**	
**Retinal diseases**		**Male**	**Female**	**18-44**	**45-64**	**≥65**	**East**	**Midwest**	**n = 313**	**Male**	**Female**	**18-44**	**45-64**	**≥65**	**East**	**Midwest**
	n = 2872	n = 1378	n = 1494	n = 250	n = 1261	n = 1361	n = 1304	n = 1568	n = 313	n = 150	n = 163	n = 33	n = 140	n = 140	n = 105	n = 208
DR	1724 (60)	827 (60)	897 (60)	177 (71)	978 (78)	569 (42)	829 (64)	895 (57)	200 (64)	95 (63)	105 (64)	22 (67)	112 (80)	66 (47)	68 (65)	132 (64)
AMD	642 (22)	341 (25)	301 (20)	0 (0)	93 (7.4)	549 (40)	287 (22)	355 (23)	52 (17)	25 (17)	27 (17)	0 (0)	6 (4.3)	46 (33)	21 (20)	31 (15)
RVO	99 (3.4)	50 (3.6)	49 (3.3)	13 (5.2)	28 (2.2)	58 (4.3)	46 (3.5)	53 (3.4)	6 (1.9)	5 (3.3)	1 (0.61)	1 (3.0)	2 (1.4)	3 (2.1)	2 (1.9)	4 (1.9)
Myopic maculopathy	159 (5.5)	59 (4.3)	100 (6.7)	23 (9.2)	82 (6.5)	54 (4.0)	57 (4.4)	102 (6.5)	20 (6.4)	7 (4.7)	13 (8.0)	3 (9.1)	6 (4.3)	11 (7.9)	6 (5.7)	14 (6.7)
RRD	20 (0.70)	11 (0.80)	9 (0.60)	3 (1.2)	10 (0.79)	7 (0.51)	8 (0.61)	12 (0.77)	3 (0.96)	2 (1.3)	1 (0.61)	1 (3.0)	1 (0.71)	1 (0.71)	1 (0.95)	2 (0.96)
ERM	35 (1.2)	10 (0.73)	25 (1.7)	1 (0.40)	3 (0.24)	31 (2.3)	10 (0.77)	25 (1.6)	3 (0.96)	2 (1.3)	1 (0.61)	0 (0)	0 (0)	3 (2.1)	1 (0.95)	2 (0.96)
Macular hole	25 (0.87)	8 (0.58)	17 (1.1)	0 (0)	8 (0.63)	17 (1.2)	12 (0.92)	13 (0.83)	2 (0.64)	2 (1.3)	0 (0)	0 (0)	0 (0)	2 (1.4)	0 (0)	2 (0.96)
CSC	5 (0.17)	3 (0.22)	2 (0.13)	3 (1.2)	2 (0.16)	0 (0)	0 (0)	5 (0.32)	0 (0)	0 (0)	0 (0)	0 (0)	0 (0)	0 (0)	0 (0)	0 (0)
Hypertensive retinopathy	9 (0.31)	3 (0.22)	6 (0.40)	2 (0.80)	1 (0.079)	6 (0.44)	2 (0.15)	7 (0.45)	0 (0)	0 (0)	0 (0)	0 (0)	0 (0)	0 (0)	0 (0)	0 (0)
RP	12 (0.42)	8 (0.58)	4 (0.27)	4 (1.6)	5 (0.40)	3 (0.22)	6 (0.46)	6 (0.38)	6 (1.9)	3 (2.0)	3 (1.8)	2 (6.1)	4 (2.9)	0 (0)	2 (1.9)	4 (1.9)
RAO	0 (0)	0 (0)	0 (0)	0 (0)	0 (0)	0 (0)	0 (0)	0 (0)	1 (0.32)	1 (0.67)	0 (0)	0 (0)	0 (0)	1 (0.71)	0 (0)	1 (0.48)
Idiopathic CNV	3 (0.10)	1 (0.073)	2 (0.13)	0 (0)	2 (0.16)	1 (0.07)	0 (0)	3 (0.19)	0 (0)	0 (0)	0 (0)	0 (0)	0 (0)	0 (0)	0 (0)	0 (0)
VMT	3 (0.10)	1 (0.073)	2 (0.13)	0 (0)	1 (0.079)	2 (0.15)	1 (0.077)	2 (0.13)	1 (0.32)	0 (0)	1 (0.61)	0 (0)	0 (0)	1 (0.71)	0 (0)	1 (0.48)
VH of unknown cause	3 (0.10)	1 (0.073)	2 (0.13)	1 (0.40)	1 (0.079)	1 (0.07)	3 (0.23)	0 (0)	0 (0)	0 (0)	0 (0)	0 (0)	0 (0)	0 (0)	0 (0)	0 (0)
ME of unknown cause	38 (1.3)	19 (1.4)	19 (1.3)	6 (2.4)	13 (1.0)	19 (1.4)	13 (1.0)	25 (1.6)	3 (0.96)	0 (0)	3 (1.8)	1 (3.0)	1 (0.71)	1 (0.71)	0 (0)	3 (1.4)
Others	95 (3.3)	36 (2.6)	59 (3.9)	17 (6.8)	34 (2.7)	44 (3.2)	30 (2.3)	65 (4.1)	16 (5.1)	8 (5.3)	8 (4.9)	3 (9.1)	8 (5.7)	5 (3.6)	4 (3.8)	12 (5.8)

BCVA – best-corrected visual acuity, DR – diabetic retinopathy, AMD – age-related macular degeneration, RVO – retinal vein occlusion, RRD – rhegmatogenous retinal detachment, ERM – epiretinal membrane, CSC – central serous chorioretinopathy, RP – retinal pigmentosa, RAO – retinal artery occlusion, CNV – choroidal neovasculariation.

*The data was presented as number (proportion).

**Figure 2:**
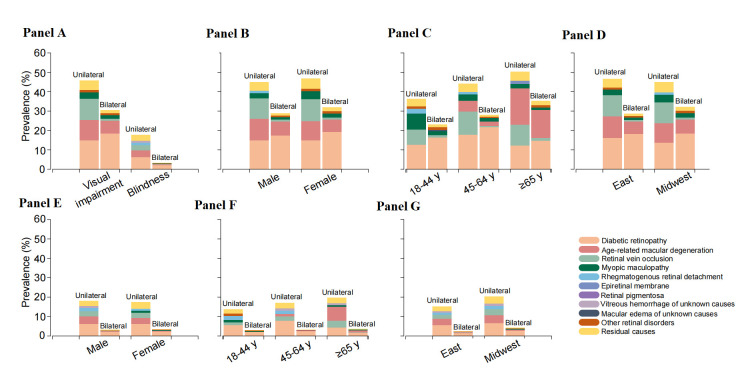
Visual burden caused by major types of retinal diseases in Chinese adults based on World Health Organization (WHO) criteria (visual impairment: best-corrected visual acuity (BCVA): <20/63-≥20/400; blindness: BCVA<20/400). **Panel A.** The contribution of major types of retinal diseases to unilateral and bilateral visual impairment for all participants. **Panel B, Panel C** and **Panel D**. The contribution of major types of retinal diseases to unilateral and bilateral visual impairment by gender (**Panel B**), age (**Panel C**), and region (**Panel D**). **Panel E, Panel F a**nd **Panel G**. The contribution of major types of retinal diseases to unilateral and bilateral blindness by gender (**Panel E**), age (**Panel F**), and region (**Panel G**). Other retinal disorders: the relative rare retinal disorders, accounting for <0.1% of total sample, are grouped in this subset.

**Figure 3:**
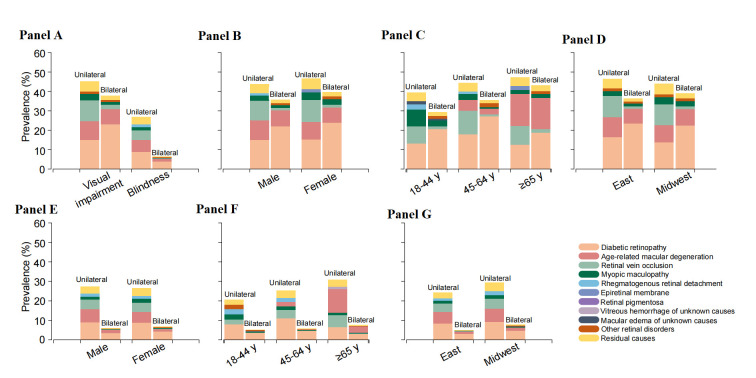
Visual burden caused by major types of retinal diseases in Chinese adults based on the USA criteria (visual impairment: best-corrected visual acuity (BCVA): <20/40-≥20/200; blindness: BCVA<20/200). **Panel A**. The contribution of major types of retinal diseases to unilateral and bilateral visual impairment for all participants. **Panel B, Panel C** and **Panel D**. The contribution of major types of retinal diseases to unilateral and bilateral visual impairment by gender (**Panel B**), age (**Panel C**), and region (**Panel D**). **Panel E, Panel F** and **Panel G**. The contribution of major types of retinal diseases to unilateral and bilateral blindness by gender (**Panel E**), age (**Panel F**), and region (**Panel G**). Other retinal disorders: the relative rare retinal disorders, accounting for <0.1% of total sample, are grouped in this subset.

For bilateral visual disabilities among patients with retinal diseases, overwhelmingly, DR was the leading cause, accounting for 60% and 64% of visual impairment and blindness, respectively. AMD (visual impairment: 22%, blindness: 17%) and myopic maculopathy (visual impairment: 5.5%, blindness: 6.4%) were the next two leading causes of bilateral vision loss. Other contributing causes included RVO (visual impairment: 3.4%, blindness: 1.9%), macular oedema of unknown causes (visual impairment: 1.3%, blindness: 0.96%), RRD (visual impairment: 0.70%, blindness: 0.96%), and epiretinal membrane (visual impairment: 1.2%, blindness: 0.96%). The age, gender specific-proportion of bilateral visual disabilities varied by cause, which is similar to unilateral vision loss (**Table 4**). The above trend continued when using the US criteria (Table S2 in the **Online Supplementary Document**). The top five predominant causes of visual impairment and blindness by age, gender and geographic regions based on WHO and US criteria are presented in **Figure 2**, panels A, B, C, D, E, F and G and **Figure 3**, panels A, B, C, D, E, F and G, respectively.

Multivariate Logistic analyses indicated that women (bilateral vision loss: *P* = 0.011), older patients (unilateral vision loss: 45-64 years: *P* < 0.001, ≥65 years: *P* < 0.001; bilateral vision loss: 45-64 years: *P* = 0.003, ≥65 years: *P* < 0.001 (reference: 18-44 years)) and those from Midwest China (unilateral and bilateral vision loss: both *P* < 0.001) were more likely to suffer from vision loss for both definitions. The details are summarised in **Table 5** and **Table 6**.

**Table 5:** The association of unilateral vision loss with gender, age and region

Variables	Univariable		Multivariable		
	**Odds ratio (95% CI)**	***P*-value**	**Regression coefficient (SE)**	**Odds ratio (95% CI)**	***P*-value**
**WHO criteria**					
Gender (female vs. male)	1.05 (1.00-1.10)	0.043	0.10 (0.03)	1.01 (0.96-1.06)	0.720
Age					
*18-44 years*	Reference		Reference		
*45-64 years*	1.59 (1.47-1.72)	<0.001*	0.46 (0.04)	1.58 (1.46-1.71)	<0.001*
*≥65 years*	2.34 (2.16-2.53)	<0.001*	0.85 (0.04)	2.34 (2.16-2.53)	<0.001*
Region (East vs. Midwest)	0.86 (0.82-0.90)	<0.001*	-0.16 (0.02)	0.85 (0.81-0.90)	<0.001*
**USA criteria**					
Gender (female vs. male)	1.10 (1.04-1.16)	<0.001*	0.05 (0.03)	1.05 (1.00-1.11)	0.074
Age					
*18-44 years*	Reference		Reference		
*45-64 years*	1.61 (1.49-1.75)	<0.001*	0.47 (0.04)	1.60 (1.48-1.74)	<0.001*
*≥65 years*	2.53 (2.33-2.75)	<0.001*	0.92 (0.04)	2.52(2.32-2.74)	<0.001*
Region (East vs. Midwest)	0.87 (0.83-0.92)	<0.001*	-0.15 (0.03)	0.86 (0.82-0.91)	<0.001*

CI – confidence interval, SE – standard error, WHO – World Health Organization

*Statistically significant.

**Table 6:** The association of bilateral vision loss with gender, age and region

Variables	Univariable		Multivariable		
	**Odds ratio (95% CI)**	***P*-value**	**Regression coefficient (SE)**	**Odds ratio (95% CI)**	***P*-value**
**WHO criteria**					
Gender (female vs. male)	1.16 (1.07-1.26)	0.001*	0.11 (0.04)	1.12 (1.03-1.22)	0.011*
Age					
*18-44 years*	Reference		Reference		
*45-64 years*	1.29 (1.11-1.50)	0.001*	0.23 (0.08)	1.26 (1.08-1.46)	0.003*
*≥65 years*	1.80 (1.55-2.10)	<0.001*	0.56 (0.08)	1.75 (1.51-2.04)	<0.001*
Region (East vs. Midwest)	0.78 (0.72-0.85)	<0.001*	-0.25 (0.04)	0.78 (0.72-0.85)	<0.001*
**US criteria**					
Gender (female vs. male)	1.21 (1.12-1.32)	<0.001*	0.15 (0.04)	1.16 (1.07-1.26)	<0.001*
Age					
*18-44 years*	Reference		Reference		
*45-64 years*	1.34 (1.16-1.54)	<0.001*	0.27 (0.07)	1.31 (1.14-1.50)	<0.001*
*≥65 years*	1.95 (1.69-2.24)	<0.001*	0.64 (0.07)	1.89 (1.64-2.17)	<0.001*
Region (East vs. Midwest)	0.80 (0.74-0.87)	<0.001*	-0.22 (0.04)	0.80 (0.74-0.87)	<0.001*

CI – confidence interval, SE – standard error, WHO – World Health Organization

*Statistically significant.

## DISCUSSION

This study first investigated the proportion of vision loss of different types of retinal diseases on a national scale. Retinal disorders cause severe visual impairment. DR, the most prevalent retinal disease, also contributed most substantially to visual disabilities, which was most evident for patients aged 18 to 64 years with retinal diseases. For patients aged 65 years or older, AMD is the most contributing factor. Female, aged patients and those from Midwest China exhibited higher rates of visual disabilities.

The threat of retinal diseases has long been signalled [1,5,22]. The data presented in this analysis are striking: among Chinese patients with retinal diseases, the proportion of unilateral visual impairment and blindness were as much as 46% and 18%, respectively. Whereas the frequency of bilateral visual impairment and blindness were 31% and 3.3%, respectively. Because the retina, as an important structure of central nervous system, contains a huge diversity of neuronal cells, unlike completely treatable causes, the visual impairment due to retinal abnormalities is hard to reverse. Therefore, the steps needed to tackle retinal disturbances, including boosting public awareness, better screening, early detection and timely intervention, have been consistently recommended.

Among patients with retinal diseases, DR is accountable for the most visual disabilities, especially for working-aged people, and AMD contributes greatest in patients 65 years or older. Consistent findings are also indicated in Africa [22]. According to the latest comprehensive assessment of global visual burden analysed in population-based studies [5], DR and AMD have been among the top five causes of vision loss [1,5]. The prevalence of blindness due to AMD declined by almost 30%, however, this rate for DR still increased by 15% through past three decades [1,5]. Possible attributes may include increasing prevalence of diabetes and high incidence of undiagnosed diabetes among young adults aged 18 to 45 years old [23,24]. This global dilemma highlights the need for improved screening and education programme, as well as better control of metabolic risk factors among individuals with diabetes and prediabetes. Some studies have suggested otherwise [3,8-10]. The data in Japan [10], China [8,16] and Netherlands [3,9] identified myopic maculopathy as the major retinal cause of vision loss. In India, retinitis pigmentosa and chorioretinitis scars were the top two causes of retinal blindness [6]. Diversified retinal causes of vision loss may be related to the differences in diagnostic procedure, screening system, ethnicity, definition of vision loss and study design. Of note, identifying risk factors and their interactions, and accurate risk stratification for population are pivotal to establish monitoring guidelines and continuous screenings [25]. In DR, outreach screening should be implemented in young adults [26,27]. In addition, sustained education on risk factor control [26] and prevention of unhealthy lifestyles [28], dietary recommendations [29,30], as well as psychosocial support [31] are also warranted for young patients with diabetes. With these measures, severe vision loss, work disability, compromised quality of life and economic burden caused by DR would have been substantially reduced [32,33]. Overall, the percentage of visual impairment and blindness caused by retinal diseases elevated with increasing age, with the exception that DR mostly affected working-aged adults, in addition, myopic maculopathy, RRD and CSC caused the greatest visual burden to even younger population. Of note, these particular retinal diseases that caused visual disabilities in more productive years of life deserves specific attention, or it might produce a large socioeconomic burden, perhaps as much as or even more than that of cataract and AMD.

Our analysis suggested a female preponderance for visual impairment and blindness among most retinal diseases. The finding is universal across different countries [1,4,7,15,16,34,35], nonetheless, the reasons underlying this gender discrepancy remain unclear.

Larger proportion of patients from Midwest China were visually impaired than those from East China. Compared to the East coastal cities in China, Midwest regions is less developed. Similar geographic variations in all-cause prevalence of vision loss were also observed [36]. This regional difference is likely driven by environmental variations, cultural diversity, compounded by inequable access to eye care services. Research efforts should be accelerated to address geographic disparity and to inform policy and practices.

Our study initially described the visual burdens caused by major types of retinal diseases from 51 centres covering mainland China. However, several points should be noted when considering the generalisability of our findings. First, visual-field constriction that would have been missed for the perimetry was not performed as part of the ocular examinations. Accordingly, the visual disabilities due to peripheral retinal disease, such as retinal pigmentosa, might have been underestimated. Second, even after two-round rechecking of medical details by independent senior investigators, the predominate cause for 546 patients with vitreous hemorrhage and 675 with macular oedema could not be exactly allocated. Third, the selection bias, although unlikely, still could not be completely excluded. In addition, possible confounders and inconsistencies across institutions and regions exist. Our study is the first such effort, however, only a continuous monitoring with a unified methodology could better reflect a true situation and provide high-level evidence for policy-making. Despite these limitations, it is noteworthy that the strengths of this study are the inclusion of a large and representative sample undergoing a comprehensive assessment of the visual impairment covering a wide spectrum of retinal disorders.

## CONCLUSIONS

Accurate estimates of the vision loss due to different types of retinal diseases are pivotal to inform optimal eye health care planning and reallocation of medical resources. Retinal disorders cause substantial visual burden in China. DR, the most prevalent retinal disease, is the leading retinal cause of visual disabilities. This fact demands that actions to tackle DR, this largely preventable global problem, should be scaled up urgently. In addition, outreach screening should be implemented to enhance equity of access among underserved groups, such as, women, the elderly and those from less developed regions.

## Additional material


Online Supplementary Document

## References

[1] Flaxman SR Bourne RRA Resnikoff S Ackland P Braithwaite T Cicinelli MV Global causes of blindness and distance vision impairment 1990-2020: a systematic review and meta-analysis. Lancet Glob Health. 2017;5:e1221-34. 10.1016/S2214-109X(17)30393-5 29032195

[2] Pérès K Matharan F Daien V Nael V Edjolo A Bourdel-Marchasson I 1st Visual Loss and Subsequent Activity Limitations in the Elderly: The French Three-City Cohort. Am J Public Health. 2017;107:564-9. 10.2105/AJPH.2016.303631 28207341 PMC5343696

[3] Ramrattan RS Wolfs RC Panda-Jonas S Jonas JB Bakker D Pols HA Prevalence and causes of visual field loss in the elderly and associations with impairment in daily functioning: the Rotterdam Study. Arch Ophthalmol. 2001;119:1788-94. 10.1001/archopht.119.12.1788 11735788

[4] GBD 2019 Blindness and Vision Impairment Collaborators; Vision Loss Expert Group of the Global Burden of Disease Study Trends in prevalence of blindness and distance and near vision impairment over 30 years: an analysis for the Global Burden of Disease Study. Lancet Glob Health. 2021;9:e130-43. 10.1016/S2214-109X(20)30425-3 33275950 PMC7820390

[5] GBD 2019 Blindness and Vision Impairment Collaborators Vision Loss Expert Group of the Global Burden of Disease Study Causes of blindness and vision impairment in 2020 and trends over 30 years, and prevalence of avoidable blindness in relation to VISION 2020: the Right to Sight: an analysis for the Global Burden of Disease Study. Lancet Glob Health. 2021;9:e144-60. 10.1016/S2214-109X(20)30489-7 33275949 PMC7820391

[6] Dandona L Dandona R Naduvilath TJ McCarty CA Nanda A Srinivas M Is current eye-care-policy focus almost exclusively on cataract adequate to deal with blindness in India? Lancet. 1998;351:1312-6. 10.1016/S0140-6736(97)09509-3 9643793

[7] Zhao J Xu X Ellwein LB Guan H He M Liu P Causes of Visual Impairment and Blindness in the 2006 and 2014 Nine-Province Surveys in Rural China. Am J Ophthalmol. 2019;197:80-7. 10.1016/j.ajo.2018.09.011 30240726

[8] Xu L Wang Y Li Y Wang Y Cui T Li J Causes of blindness and visual impairment in urban and rural areas in Beijing: the Beijing Eye Study. Ophthalmology. 2006;113:1134.e1-11. 10.1016/j.ophtha.2006.01.035 16647133

[9] Klaver CC Wolfs RC Vingerling JR Hofman A de Jong PT Age-specific prevalence and causes of blindness and visual impairment in an older population: the Rotterdam Study. Arch Ophthalmol. 1998;116:653-8. 10.1001/archopht.116.5.653 9596502

[10] Iwase A Araie M Tomidokoro A Yamamoto T Shimizu H Kitazawa Y Prevalence and causes of low vision and blindness in a Japanese adult population: the Tajimi Study. Ophthalmology. 2006;113:1354-62. 10.1016/j.ophtha.2006.04.022 16877074

[11] Rogers S McIntosh RL Cheung N Lim L Wang JJ Mitchell P The prevalence of retinal vein occlusion: pooled data from population studies from the United States, Europe, Asia, and Australia. Ophthalmology. 2010;117:313-9 e1. 10.1016/j.ophtha.2009.07.017 20022117 PMC2945292

[12] Mitchell P Smith W Chang A Prevalence and associations of retinal vein occlusion in Australia. The Blue Mountains Eye Study. Arch Ophthalmol. 1996;114:1243-7. 10.1001/archopht.1996.01100140443012 8859084

[13] Saw SM Foster PJ Gazzard G Seah S Causes of blindness, low vision, and questionnaire-assessed poor visual function in Singaporean Chinese adults: The Tanjong Pagar. Ophthalmology 2004;111:1161-8. 10.1016/j.ophtha.2003.09.040 15177966

[14] Zheng Y Lavanya R Wu R Wong WL Wang JJ Mitchell P Prevalence and causes of visual impairment and blindness in an urban Indian population: the Singapore Indian Eye Study. Ophthalmology. 2011;118:1798-804. 10.1016/j.ophtha.2011.02.014 21621261

[15] Wong TY Chong EW Wong WL Rosman M Aung T Loo JL Prevalence and causes of low vision and blindness in an urban malay population: the Singapore Malay Eye Study. Arch Ophthalmol. 2008;126:1091-9. 10.1001/archopht.126.8.1091 18695104

[16] Tang Y Wang X Wang J Huang W Gao Y Luo Y Prevalence and Causes of Visual Impairment in a Chinese Adult Population: The Taizhou Eye Study. Ophthalmology. 2015;122:1480-8. 10.1016/j.ophtha.2015.03.022 25986897

[17] Muñoz B West SK Rubin GS Schein OD Quigley HA Bressler SB Causes of blindness and visual impairment in a population of older Americans: The Salisbury Eye Evaluation Study. Arch Ophthalmol. 2000;118:819-25. 10.1001/archopht.118.6.819 10865321

[18] Zhao J Ellwein LB Cui H Ge J Guan H Lv J Prevalence of vision impairment in older adults in rural China: the China Nine-Province Survey. Ophthalmology. 2010;117:409-16. 10.1016/j.ophtha.2009.11.023 20079923 PMC6029941

[19] Early Treatment Diabetic Retinopathy Study Research G Grading Diabetic Retinopathy from Stereoscopic Color Fundus Photographs - An Extension of the Modified Airlie House Classification: ETDRS Report Number 10. Ophthalmology. 2020;127 4S:S99-119. 10.1016/j.ophtha.2020.01.030 32200833

[20] Klein R Davis MD Magli YL Segal P Klein BE Hubbard L The Wisconsin age-related maculopathy grading system. Ophthalmology. 1991;98:1128-34. 10.1016/S0161-6420(91)32186-9 1843453

[21] Ruiz-Medrano J Montero JA Flores-Moreno I Arias L Garcia-Layana A Ruiz-Moreno JM Myopic maculopathy: Current status and proposal for a new classification and grading system (ATN). Prog Retin Eye Res. 2019;69:80-115. 10.1016/j.preteyeres.2018.10.005 30391362

[22] Onakpoya OH Udonwa P Awe OO The Burden of Visual Impairment and Blindness from Vitreoretinal Diseases: A Nigerian Tertiary Hospital Retina Unit Experience. Niger Med J. 2020;61:257-61. 10.4103/nmj.NMJ_210_16 33487849 PMC7808290

[23] Wang L Li X Wang Z Bancks MP Carnethon MR Greenland P Trends in Prevalence of Diabetes and Control of Risk Factors in Diabetes Among US Adults, 1999-2018. JAMA. 2021;326:1-13. 10.1001/jama.2021.9883 34170288 PMC8233946

[24] Wang L Peng W Zhao Z Zhang M Shi Z Song Z Prevalence and Treatment of Diabetes in China, 2013-2018. JAMA. 2021;326:2498-506. 10.1001/jama.2021.22208 34962526 PMC8715349

[25] Bucan K Lukic M Bosnar D Kopic A Jukic T Konjevoda S Analysis of association of risk factors for age-related macular degeneration. Eur J Ophthalmol. 2022;32:410-6. 10.1177/1120672121998900 33660548

[26] Wang L Li XG Wang ZX Bancks MP Carnethon MR Greenland P Trends in Prevalence of Diabetes and Control of Risk Factors in Diabetes Among US Adults, 1999-2018. JAMA. 2021;326:1-13. 10.1001/jama.2021.9883 34170288 PMC8233946

[27] Arslanian S Bacha F Grey M Marcus MD White NH Zeitler P Evaluation and Management of Youth-Onset Type 2 Diabetes: A Position Statement by the American Diabetes Association. Diabetes Care. 2018;41:2648-68. 10.2337/dci18-0052 30425094 PMC7732108

[28] Liu G Pan A Hu Y Chen S Qian F Rimm EB Adherence to a Healthy Lifestyle in Association With Microvascular Complications Among Adults With Type 2 Diabetes. JAMA Netw Open. 2023;6:e2252239. 10.1001/jamanetworkopen.2022.52239 36701156 PMC9880795

[29] Sasaki M Kawasaki R Rogers S Man REK Itakura K Xie J The Associations of Dietary Intake of Polyunsaturated Fatty Acids With Diabetic Retinopathy in Well-Controlled Diabetes. Invest Ophthalmol Vis Sci. 2015;56:7473-9. 10.1167/iovs.15-17485 26595607

[30] Ganesan S Raman R Kulothungan V Sharma T Influence of dietary-fibre intake on diabetes and diabetic retinopathy: Sankara Nethralaya-Diabetic Retinopathy Epidemiology and Molecular Genetic Study (report 26). Clin Exp Ophthalmol. 2012;40:288-94. 10.1111/j.1442-9071.2011.02594.x 21575120

[31] Grey M Sullivan-Bolyai S Boland EA Tamborlane WV Yu C Personal and family factors associated with quality of life in adolescents with diabetes. Diabetes Care. 1998;21:909-14. 10.2337/diacare.21.6.909 9614606

[32] Png ME Yoong J Phan TP Wee HL Current and future economic burden of diabetes among working-age adults in Asia: conservative estimates for Singapore from 2010-2050. BMC Public Health. 2016;16:153. 10.1186/s12889-016-2827-1 26880337 PMC4754926

[33] Von Korff M Ludman E Katon W Oliver M Lin EHB Rutter C Work disability among individuals with diabetes. Diabetes Care. 2005;28:1326-32. 10.2337/diacare.28.6.1326 15920047

[34] Murthy GV Gupta S Ellwein LB Munoz SR Bachani D Dada VK A population-based eye survey of older adults in a rural district of Rajasthan: I. Central vision impairment, blindness, and cataract surgery. Ophthalmology. 2001;108:679-85. 10.1016/S0161-6420(00)00579-0 11297483

[35] Klein R Klein BE Linton KL De Mets DL The Beaver Dam Eye Study: visual acuity. Ophthalmology. 1991;98:1310-5. 10.1016/S0161-6420(91)32137-7 1923372

[36] Cheng JW Cheng SW Cai JP Li Y Wei RL The prevalence of visual impairment in older adults in mainland China: a systematic review and meta-analysis. Ophthalmic Res. 2013;49:1-10. 10.1159/000327144 22965304

