# Supplementary material for: Visual impairment and blindness caused by retinal diseases: A nationwide register-based study
Source: J Glob Health. 2023 Nov 3;13:04126. doi: 10.7189/jogh.13.04126 (PMC10623496; doi:10.7189/jogh.13.04126)
Supplement: Online Supplementary Document [file jogh-13-04126-s001.pdf]

ONLINE SUPPLEMENTARY DOCUMENT

Title: Visual impairment and blindness caused by retinal diseases: A nationwide register-based study

Authors: Chuandi Zhou, Shu Li, Luyao Ye, Chong Chen, Shu Liu, Hongxia Yang, Peng Zhuang, Zengye Liu, Hongwen Jiang, Jing Han, Yiping Jiang, Liqin Zhou, Xiyuan Zhou, Jun Xiao, Cangxia Zhang, Lihui Wen, Changjun Lan, Yuqing Wang, Tao Sun, Li Jiang, Peipei Xie, Fan Chen, Ge Liang, Dongdong Fu, Tianzi Zhang, Xuetao Shi, Zhengyu Song, Xinghong Liu, Shanshan Li, Pengcheng Li, Xiangzhou Xu, Qinfeng Wei, Weibang Wang, Xionggao Huang, Zhen De, Aijun Deng, Lin Ding, Xiuhong Pan, Haiyan Wen, Zhongchen Zhang, Hongbin Lv, Jian Zhang, Xuemin Tian, Zhen Deng, Hua Wang, Fang Wang, Yu Wang, Hongchao Zhao, Yanhong Fang, Yuyu Wu, Yufeng Wu, Nian Shen, Bo Li, Xiaorong Li, Hong Dai, Naiqing Zhao, Xiaodong Sun, Zhi Zheng, Kun Liu, Xun Xu

Supplementary material summary

Contents	Numbered pages
Table S1. Proportional retinal causes of unilateral visual impairment and blindness by age, gender and regions based on the United States criteria	2
Table S2. Proportional retinal causes of bilateral visual impairment and blindness by age, gender and regions based on the United States criteria	3

Table S1. Proportional retinal causes of unilateral visual impairment and blindness by age, gender and regions based on the United States criteria

Retinal diseases	Visual impairment (BCVA, <20/40-≥20/200)								Blindness (BCVA, <20/200)							
	Total	Gender		Age (yrs)			Regions		Total	Gender		Age (yrs)			Regions	
		Male	Female	18-44	45-64	≥65	East	Midwest		Male	Female	18-44	45-64	≥65	East	Midwest
	(n=12803)	(n=6263)	(n=6540)	(n=1253)	(n=5866)	(n=5684)	(n=6506)	(n=6297)	(n=7630)	(n=3917)	(n=3713)	(n=616)	(n=3334)	(n=3680)	(n=3409)	(n=4221)
DR	4268(33)	2136(34)	2132(33)	421(34)	2352(40)	1495(26)	2306(35)	1962(31)	2514(33)	1278(33)	1236(33)	253(41)	1470(44)	791(21)	1189(35)	1325(31)
AMD	2719(21)	1442(23)	1277(20)	0(0)	752(13)	1967(35)	1440(22)	1279(20)	1764(23)	983(25)	781(21)	0(0)	313(9.4)	1451(39)	809(24)	955(23)
RVO	3049(24)	1459(23)	1590(24)	278(22)	1603(27)	1168(21)	1525(23)	1524(24)	1368(18)	712(18)	656(18)	81(13)	568(17)	719(20)	623(18)	745(18)
Myopic maculopathy	909(7.1)	372(5.9)	537(8.2)	277(22)	395(6.7)	237(4.2)	370(5.7)	539(8.6)	472(6.2)	185(4.7)	287(7.7)	84(14)	226(6.8)	162(4.4)	204(6.0)	268(6.3)
RRD	328(2.6)	184(2.9)	144(2.2)	81(6.5)	184(3.1)	63(1.1)	158(2.4)	170(2.7)	442(5.8)	254(6.5)	188(5.1)	78(13)	253(7.6)	111(3.0)	154(4.5)	288(6.8)
ERM	349(2.7)	121(1.9)	228(3.5)	6(0.48)	89(1.5)	254(4.5)	163(2.5)	186(3.0)	73(0.96)	26(0.66)	47(1.3)	1(0.16)	18(0.54)	54(1.5)	24(0.70)	49(1.2)
Macular hole	204(1.6)	70(1.1)	134(2.0)	12(0.96)	86(1.5)	106(1.9)	114(1.8)	90(1.4)	118(1.5)	33(0.84)	85(2.3)	5(0.81)	54(1.6)	59(1.6)	55(1.6)	63(1.5)
CSC	70(0.55)	56(0.89)	14(0.21)	25(2.0)	41(0.70)	4(0.070)	35(0.54)	35(0.56)	7(0.092)	5(0.13)	2(0.054)	1(0.16)	5(0.15)	1(0.027)	3(0.088)	4(0.095)
Hypertensive retinopathy	76(0.59)	42(0.67)	34(0.52)	12(0.96)	24(0.41)	40(0.70)	38(0.58)	38(0.60)	26(0.34)	15(0.38)	11(0.30)	5(0.81)	10(0.30)	11(0.30)	18(0.53)	8(0.19)
RP	26(0.20)	15(0.24)	11(0.17)	11(0.88)	9(0.15)	6(0.11)	12(0.18)	14(0.22)	23(0.30)	12(0.31)	11(0.30)	5(0.81)	15(0.45)	3(0.082)	8(0.23)	15(0.36)
RAO	10(0.078)	6(0.096)	4(0.061)	1(0.080)	5(0.085)	4(0.070)	2(0.031)	8(0.13)	60(0.79)	40(1.0)	20(0.54)	2(0.32)	31(0.93)	27(0.73)	30(0.88)	30(0.71)
Idiopathic CNV	13(0.10)	4(0.064)	9(0.14)	9(0.72)	3(0.051)	1(0.018)	3(0.046)	10(0.16)	5(0.066)	2(0.051)	3(0.081)	2(0.32)	2(0.060)	1(0.027)	1(0.029)	4(0.095)
VMT	19(0.15)	9(0.14)	10(0.15)	2(0.16)	3(0.051)	14(0.25)	10(0.15)	9(0.14)	4(0.052)	1(0.026)	3(0.081)	0(0)	1(0.030)	3(0.082)	2(0.059)	2(0.047)
VH of unknown cause	130(1.0)	62(0.99)	68(1.0)	13(1.0)	76(1.3)	41(0.72)	49(0.75)	81(1.3)	335(4.4)	178(4.5)	157(4.2)	27(4.4)	177(5.3)	131(3.6)	148(4.3)	187(4.4)
ME of unknown cause	245(1.9)	121(1.9)	124(1.9)	56(4.5)	84(1.4)	105(1.8)	107(1.6)	138(2.2)	159(2.1)	79(2.0)	80(2.2)	32(5.2)	71(2.1)	56(1.5)	34(1.0)	125(3.0)
Others	388(3.0)	164(2.6)	224(3.4)	49(3.9)	160(2.7)	179(3.1)	174(2.7)	214(3.4)	260(3.4)	114(2.9)	146(3.9)	40(6.5)	120(3.6)	100(2.7)	107(3.1)	153(3.6)

BCVA: best-corrected visual acuity; DR: diabetic retinopathy; AMD: age-related macular degeneration; RVO: retinal vein occlusion; RRD: rhegmatogenous retinal detachment; ERM: epiretinal membrane; CSC: central serous chorioretinopathy; RP: retinal pigmentosa; RAO: retinal artery occlusion; CNV: choroidal neovascularization; VMT: vitreous macular traction; VH: vitreous hemorrhage; ME: macular edema.

Table S2. Proportional retinal causes of bilateral visual impairment and blindness by age, gender and regions based on the United States criteria

Retinal diseases	Visual impairment (BCVA, <20/40-≥20/200)								Blindness (BCVA, <20/200)							
	Total	Gender		Age (yrs)			Regions		Total	Gender		Age (yrs)			Regions	
		Male	Female	18-44	45-64	≥65	East	Midwest		Male	Female	18-44	45-64	≥65	East	Midwest
	(n=3570)	(n=1707)	(n=1863)	(n=317)	(n=1590)	(n=1663)	(n=1666)	(n=1904)	(n=610)	(n=289)	(n=321)	(n=57)	(n=262)	(n=291)	(n=227)	(n=383)
DR	2164(61)	1047(61)	1117(60)	223(70)	1220(77)	721(43)	1072(64)	1092(57)	367(60)	166(57)	201(63)	41(72)	208(79)	118(41)	136(60)	231(60)
AMD	752(21)	386(23)	366(20)	0(0)	131(8.2)	621(37)	339(20)	413(22)	133(22)	75(26)	58(18)	0(0)	9(3.4)	124(43)	55(24)	78(20)
RVO	125(3.5)	63(3.7)	62(3.3)	16(5.0)	38(2.4)	71(4.3)	56(3.4)	69(3.6)	14(2.3)	7(2.4)	7(2.2)	1(1.8)	5(1.9)	8(2.7)	9(4.0)	5(1.3)
Myopic maculopathy	204(5.7)	71(4.2)	133(7.1)	33(10)	95(6.0)	76(4.6)	72(4.3)	132(6.9)	35(5.7)	15(5.2)	20(6.2)	5(8.8)	15(5.7)	15(5.2)	13(5.7)	22(5.7)
RRD	22(0.62)	15(0.9)	7(0.38)	4(1.3)	12(0.75)	6(0.36)	12(0.72)	10(0.53)	5(0.82)	2(0.69)	3(0.93)	1(1.8)	2(0.76)	2(0.69)	1(0.44)	4(1.0)
ERM	51(1.4)	17(1.0)	34(1.8)	1(0.32)	5(0.31)	45(2.7)	14(0.84)	37(1.9)	5(0.82)	4(1.4)	1(0.31)	0(0)	0(0)	5(1.7)	1(0.44)	4(1.0)
Macular hole	29(0.81)	11(0.64)	18(1.0)	1(0.32)	11(0.69)	17(1.0)	14(0.84)	15(0.79)	6(1.0)	4(1.4)	2(0.62)	0(0)	1(0.38)	5(1.7)	3(1.3)	3(0.78)
CSC	7(0.20)	5(0.29)	2(0.11)	3(0.95)	4(0.25)	0(0)	1(0.060)	6(0.32)	0(0)	0(0)	0(0)	0(0)	0(0)	0(0)	0(0)	0(0)
Hypertensive retinopathy	22(0.62)	9(0.53)	13(0.70)	5(1.6)	4(0.25)	13(0.78)	8(0.48)	14(0.74)	1(0.16)	0(0)	1(0.31)	0(0)	0(0)	1(0.34)	1(0.44)	0(0)
RP	17(0.48)	10(0.59)	7(0.38)	5(1.6)	8(0.50)	4(0.24)	9(0.54)	8(0.42)	6(1.0)	3(1.0)	3(0.93)	2(3.5)	4(1.5)	0(0)	2(0.88)	4(1.0)
RAO	1(0.028)	0(0)	1(0.054)	0(0)	0(0)	1(0.060)	1(0.060)	0(0)	1(0.16)	1(0.35)	0(0)	0(0)	0(0)	1(0.34)	0(0)	1(0.26)
Idiopathic CNV	2(0.056)	1(0.059)	1(0.054)	0(0)	1(0.06)	1(0.060)	0(0)	2(0.11)	1(0.16)	0(0)	1(0.31)	0(0)	1(0.38)	0(0)	0(0)	1(0.26)
VMT	4(0.11)	1(0.059)	3(0.16)	0(0)	1(0.06)	3(0.18)	2(0.12)	2(0.11)	1(0.16)	0(0)	1(0.31)	0(0)	0(0)	1(0.34)	0(0)	1(0.26)
VH of unknown cause	4(0.11)	1(0.059)	3(0.16)	1(0.32)	2(0.13)	1(0.060)	2(0.12)	2(0.11)	1(0.16)	1(0.35)	0(0)	0(0)	0(0)	1(0.34)	1(0.44)	0(0)
ME of unknown cause	53(1.5)	24(1.4)	29(1.6)	9(2.8)	17(1.1)	27(1.6)	20(1.2)	33(1.7)	7(1.1)	1(0.35)	6(1.9)	2(3.5)	4(1.5)	1(0.34)	0(0)	7(1.8)
Others	113(3.2)	46(2.7)	67(3.6)	16(5.0)	41(2.6)	56(3.4)	44(2.6)	69(3.6)	27(4.4)	10(3.5)	17(5.3)	5(8.8)	13(5.0)	9(3.1)	5(2.2)	22(5.7)

BCVA: best-corrected visual acuity; DR: diabetic retinopathy; AMD: age-related macular degeneration; RVO: retinal vein occlusion; RRD: rhegmatogenous retinal detachment; ERM: epiretinal membrane; CSC: central serous chorioretinopathy; RP: retinal pigmentosa; RAO: retinal artery occlusion; CNV: choroidal neovascularization; VMT: vitreous macular traction; VH: vitreous hemorrhage; ME: macular edema.
